# Whole-Genome Sequence of the Metastatic PC3 and LNCaP Human Prostate Cancer Cell Lines

**DOI:** 10.1534/g3.117.039909

**Published:** 2017-04-14

**Authors:** Inge Seim, Penny L. Jeffery, Patrick B. Thomas, Colleen C. Nelson, Lisa K. Chopin

**Affiliations:** * Comparative and Endocrine Biology Laboratory, Translational Research Institute-Institute of Health and Biomedical Innovation, Queensland University of Technology, Woolloongabba, Brisbane, Queensland 4102, Australia; † Australian Prostate Cancer Research Centre - Queensland, Princess Alexandra Hospital, Queensland University of Technology, Woolloongabba, Brisbane, Queensland 4102, Australia; ‡ Ghrelin Research Group, Translational Research Institute-Institute of Health and Biomedical Innovation, Queensland University of Technology, Woolloongabba, Brisbane, Queensland 4102, Australia

**Keywords:** prostate cancer, human, cell line, WGS, sequencing, genomics, Genome Report

## Abstract

The bone metastasis-derived PC3 and the lymph node metastasis-derived LNCaP prostate cancer cell lines are widely studied, having been described in thousands of publications over the last four decades. Here, we report short-read whole-genome sequencing (WGS) and *de novo* assembly of PC3 (ATCC CRL-1435) and LNCaP (clone FGC; ATCC CRL-1740) at ∼70 × coverage. A known homozygous mutation in *TP53* and homozygous loss of *PTEN* were robustly identified in the PC3 cell line, whereas the LNCaP cell line exhibited a larger number of putative inactivating somatic point and indel mutations (and in particular a loss of stop codon events). This study also provides preliminary evidence that loss of one or both copies of the tumor suppressor Capicua (*CIC*) contributes to primary tumor relapse and metastatic progression, potentially offering a treatment target for castration-resistant prostate cancer (CRPC). Our work provides a resource for genetic, genomic, and biological studies employing two commonly-used prostate cancer cell lines.

Cultured cancer cell lines, such as the human-derived PC3 and LNCaP, are critical for prostate cancer research. The androgen-dependent LNCaP cell line (clone FGC) is derived from a lymph node metastasis (Horoszewicz 1980; Horoszewicz *et al.* 1983), and the androgen-independent PC3 cell line is derived from a bone metastasis (Kaighn *et al.* 1979). Since their development, almost 40 yr ago, they have emerged as major tools in prostate cancer research (with PubMed searches 15.01.17 for “PC3 AND prostate” and “LNCaP AND prostate” returning 3266 and 7080 hits, respectively). While these cell lines have been interrogated using array- and sequencing-based technologies (Liu *et al.* 2008; Barretina *et al.* 2012; Spans *et al.* 2012; Klijn *et al.* 2015), whole-genome sequences for the PC3 and LNCaP cell lines have not been published. Albeit currently relatively costly, WGS offers better coverage than exome sequencing, and improved detection of single nucleotide and small indel mutations and structural variants such as copy number alterations (Meynert *et al.* 2014; Belkadi *et al.* 2015; Warr *et al.* 2015).

## Materials and Methods

### Cell lines

PC3 (ATCC CRL-1435) and LNCaP clone FGC (ATCC CRL-1740; hereafter termed LNCaP) prostate cancer cell lines were obtained from the American Type Culture Collection (ATCC, Rockville, MD), and maintained in Roswell Park Memorial Institute RPMI 1640 medium (Invitrogen, Carlsbad, CA) with 10% Fetal Calf Serum (Thermo Fisher Scientific, Waltham, MA), supplemented with 100 U/ml penicillin G and 100 ng/ml streptomycin (Invitrogen). All cell lines were passaged at 2–3-d intervals on reaching 70% confluency using TrypLE Select (Invitrogen). Cell morphology and viability were monitored by microscopic observation and regular *Mycoplasma* testing was performed (Universal *Mycoplasma* Detection Kit; ATCC).

### Sequencing

DNA was extracted using a QIAamp DNA mini kit (QIAGEN, Hilden, Germany) from low passage (passage four) PC3 and LNCaP cell lines, cultivated from frozen stocks obtained directly from the ATCC. Sequencing was performed by Macrogen (Seoul, South Korea). Briefly, library preparation was performed using a TruSeq Nano DNA kit (Illumina, San Diego, CA) with a target insert size of 350 bp. Paired-end libraries (150 bp) were sequenced using a HiSeqX sequencer (Illumina). Base calls were converted into FASTQ files using bcl2fastq v2.15.0 and provided to our laboratory.

### Normal prostate data

Raw data from normal human prostate samples were obtained from the National Institutes of Health’s Cancer Genome Atlas (The Cancer Genome Atlas Research Network 2015) (NCBI dbGaP: phs000178.v9.p8). These included a WGS sample (PCAWG.e22e63de-c436-43c0-a595-022622c1fe06) and three RNA-seq samples (120215-UNC10-SN254-0327-AC0CMCACXX-ACTTGA-L005, 120502-UNC14-SN744-0235-BD0YUTACXX-ACTTGA-L005, and 130221-UNC9-SN296-0338-BC1PYCACXX-TGACCA-L008). The WGS file (101 bp paired-end library; 950 M reads) was provided as an unaligned BAM (uBAM) file and converted to FASTQ files using bamUtils v1.0.14 genome.sph.umich.edu/wiki/BamUtil.

### Data processing

Raw reads (FASTQ) were trimmed using scythe v0.994 github.com/vsbuffalo/scythe, with default settings, to remove low quality bases and read-pairs, and contaminating adapter sequences.

#### Mapping of genome reads to reference genome:

FASTQ files were mapped to human reference genome GRCh38 build 82 (the reference genome in all subsequent analyses) using BWA-MEM (Li 2013), available in v0.7.12-r1039, and a sorted BAM file was generated by SAMtools v1.3.1 (Li *et al.* 2009). Genome coverage was estimated using QualiMap v2.2.1 (García-Alcalde *et al.* 2012; Okonechnikov *et al.* 2016).

#### de novo genome assembly:

PC3 and LNCaP genomes were assembled *de novo* using SGA v0.10.15 (Simpson and Durbin 2012) (available at github.com/jts/sga), as outlined in the user manual, except that assembled contigs were indexed using BWA-MEM (Li 2013) instead of the bundled Python script sga-align (calls BWA sample: bwa mem -t $CPU final-contigs.fa $READ1 $READ2 | samtools view -F2304 -b -o reads.bam -).

Resulting scaffolds were gap filled using “sga gapfill” and error-corrected FASTQ reads. The genome assemblies (gapfilled scaffolds) were evaluated using QUAST v4.3 (Gurevich *et al.* 2013) and the human reference genome. Genes of interest were interrogated in the assembled genomes using BLAST, via a local instance of SequenceServer v1.0.9 (Priyam *et al.* 2015), and GMAP v2016-11-07 (a genomic mapping and alignment program for mRNA and EST sequences) (Wu and Watanabe 2005).

#### Single nucleotide variant (SNV) and short indel calling:

Samtools v1.3.1 mpileup and bcftools (Li *et al.* 2009) were used to interrogate indexed BAM files of reads aligned to the reference genome and generate a VCF (Variant Call Format) file of SNVs and short indel variants. Variants (likely to be common germline variants) present in HapMap (Gibbs *et al.* 2003), 1000 genomes phase 3 (2504 human genomes) (Sudmant *et al.* 2015), and the National Heart Lung and Blood Institutes Exome Sequencing Project (Tennessen *et al.* 2012) (bundled variant data file available at https://goo.gl/mEogvD) were excluded.

Next, variant files (VCF) were filtered using SnpSift (Cingolani *et al.* 2012a) with the following parameters: QUAL ≥ 200 && DP ≥ 30; where QUAL denotes minimum variance confidence and DP total depth threshold. Filtered variants were annotated using SnpEff v4.3g (Cingolani *et al.* 2012b).

#### Copy number variation (CNV) calling:

To screen PC3 and LNCaP genomes for CNV, we employed the R package “cn.mops” (Copy Number estimation by a Mixture Of PoissonS) (Klambauer *et al.* 2012). Briefly, paired-end genome reads from PC3 and LNCaP were aligned to the reference genome and compared with normal prostate reads to obtain genome-wide read-depth profiles. Custom R scripts were used to parse the output.

#### Gene expression potential analysis:

We interrogated publicly available transcriptome data from PC3 (Wang *et al.* 2015) (NCBI GEO: GSE65112) and LNCaP cells (Metzger *et al.* 2016) (NCBI GEO: GSE64529). In addition, transcriptome data from normal prostate samples were obtained from TCGA (see above). Briefly, paired-end reads were trimmed using scythe, and aligned to human reference genome GRCh38 build 82 using the spliced-read mapper TopHat (v2.0.9) (Kim *et al.* 2013) and reference gene annotations to guide the alignment. Raw gene counts were computed from the generated BAM files by featureCounts v1.4.5-p1 (Liao *et al.* 2014), counting exon features of the gene annotation file (gtf) in order to include noncoding RNA genes. FeatureCounts output files were analyzed using the R programming language (v.3.2.2) (R Core Team 2013). Raw counts were normalized by Trimmed Mean of *M*-values (TMM) correction (Robinson and Oshlack 2010; Robinson *et al.* 2010). The expression of genes in normal prostate, LNCaP, and PC3 was assessed using the Universal exPression Code (UPC) method (Piccolo *et al.* 2013), available in the R package “SCAN.UPC”. This method estimates the active/inactive state of genes in a sample, where a UPC value > 0.5 indicates that a gene is actively transcribed.

#### cBioPortal analysis:

Data on copy number alterations in prostate cancer tumor tissue were obtained using the cBioPortal tool (www.cbioportal.org) (Cerami *et al.* 2012; Gao *et al.* 2013) with the following parameters: “GENE: HOMDEL HETLOSS;”, where “GENE” denotes a gene symbol. Clinical information was also downloaded and the data further analyzed using custom R scripts.

The following cBioPortal prostate cancer data sets were interrogated: ‘NEPC (Trento/Cornell/Broad 2016)’ (34 primary and 73 metastatic tumors) (Beltran *et al.* 2016), ‘Prostate (Broad/Cornell 2013)’ (55 primary tumors and 1 metastatic tumor) (Baca *et al.* 2013), ‘Prostate (FHCRC, 2016)’ (19 primary and 130 metastatic tumors) (Kumar *et al.* 2016), ‘Prostate (MICH), (11 primary and 47 metastatic tumors) (Grasso *et al.* 2012), ‘Prostate (MSKCC 2010)’ (157 primary and 37 metastatic tumors) (Taylor *et al.* 2010), ‘Prostate (MSKCC 2014)’ (101 primary and 3 metastatic tumors), (Hieronymus *et al.* 2014), ‘Prostate (SU2C)’ (150 metastatic tumors) (Robinson *et al.* 2015), and ‘Prostate (TCGA)’ (492 primary and 1 metastatic tumors) (The Cancer Genome Atlas Research Network 2015).

#### Kaplan–Meier survival analysis:

Kaplan–Meier survival analysis was performed to compare disease-free survival (DFS) in patient groups stratified by CNVs. DFS is defined as the time to either recurrence or relapse, second cancer, or death (Gill and Sargent 2006). In the context of prostate cancer, DFS is a suitable surrogate for overall survival (OS), given that metastatic disease is not curable and recurrence of disease would be expected to contribute significantly to mortality.

‘The Prostate (MSKCC 2010)’, ‘Prostate (MSKCC 2014)’, and ‘Prostate (TCGA)’ cBioPortal data sets were interrogated. Kaplan–Meier survival analysis (Rich *et al.* 2010) was performed using the R package “survival” (Therneau 2013), fitting survival curves (survfit) and computing log-rank *P*-values using the survdiff function, with *ρ* = 0 (equivalent to the method employed by UCSC Xena; see goo.gl/4knf62). Survival curves were plotted where survival was significantly different between two groups (log-rank *P* ≤ 0.05). Groups with <10 samples with recorded events were considered unreliable (Mallett *et al.* 2010).

### Gene ontology term enrichment analysis

Gene enrichment analyses were performed using DAVID (Database for Annotation, Visualization and Integrated Discovery) (Huang *et al.* 2009a). All gene groups are potentially informative, despite lower rankings, and serve to guide biological interpretation (Huang *et al.* 2009b).

### Data availability

The genome reads reported in this paper have been deposited in the BioProject database as PRJNA361315 (PC3) and PRJNA361316 (LNCaP). Code used to generate the data and CNV analysis output files (tabulated text files) are available at github.com/sciseim/PCaWGS. Genome assemblies (FASTA format) (Seim 2017a,b), and filtered and annotated single-nucleotide and indel variation data files (VCF format) (Seim 2017c), have been deposited at Zenodo. A BLAST server is available at http://ghrelinlab.org. 

## Results and Discussion

### WGS

**Figure 1:**
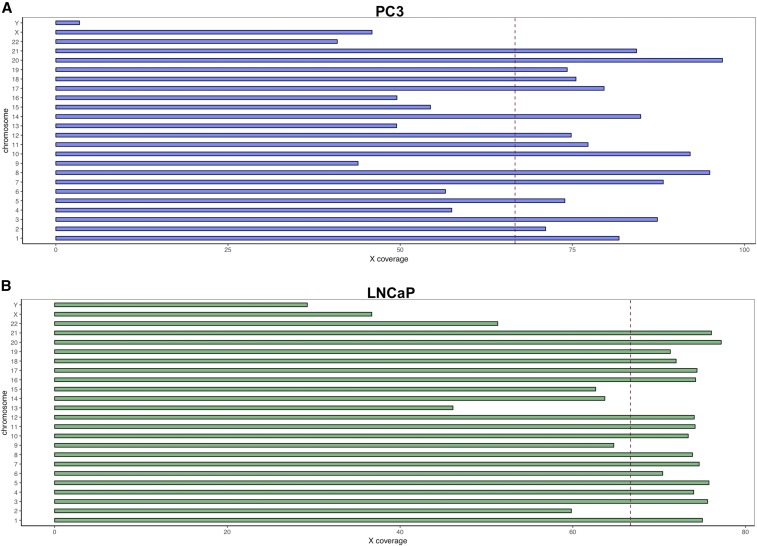
Read-depth across chromosomes in the (A) PC3 and (B) LNCaP prostate cancer cell lines. The red dotted line indicates mean genome-wide sequencing coverage (X).

PC3 and LNCaP prostate cancer cells were obtained directly from ATCC, cultured for four passages, and 150 bp paired-end reads obtained using an Illumina HiSeqX sequencer. Following read trimming, 1.53 billion reads from PC3 were retained, of which 99.9% could be aligned to the Ensembl GRCh38.82 human reference genome at ∼71 × mean coverage (Figure 1A). Similarly, we obtained 1.49 billion trimmed reads from LNCaP with a 99.9% alignment rate and mean coverage of ∼69 × (Figure 1B).

We also performed *de novo* genome assembly to allow characterization of whole-gene loci by BLAST and other mappers. The final, gap-filled PC3 genome assembly consisted of 1.66 M scaffolds (largest scaffold 692.4 kb) with an N50 of 23.3 kb and an NG50 (number of sequences with lengths equal to or larger than N50) of 22.4 kb. The LNCaP assembly consisted of 1.70 M scaffolds (largest scaffold 536.0 kb) at an N50 value of 44.4 kb and NG50 of 45.0 kb.

### Single-nucleotide and indel variation

After excluding common germline sequence variants (SNVs and short indels), filtering by SnpSift (Cingolani *et al.* 2012a), and annotation by SnpEff (Cingolani *et al.* 2012b), we identified in LNCaP 0.94 M and in PC3 0.56 M sequence variants (SNVs and short indels) that were private or unique to the particular cell line (Table 1). As expected, the majority of variants were found in noncoding regions.

**Table 1:** SNV and indel variant-calling statistics of the prostate cancer cell lines PC3 and LNCaP

	PC3 Private		LNCaP Private		Shared	
Number and percentage of variants by type						
SNVs	318,380	34.0%	404,282	72.1%	166,912	65.0%
Indels	618,149	66.0%	156,182	27.9%	89,919	35.0%
Number of events by type						
3′-UTR	15,572		10,500		3938	
5′-UTR premature start codon	211		289		49	
5′-UTR	2613		1868		692	
Conservative_inframe_deletion	39		22		7	
Conservative_inframe_insertion	468		119		72	
Disruptive_inframe_deletion	62		19		7	
Disruptive_inframe_insertion	172		64		44	
Downstream_gene	107,761		56,728		29,770	
Frameshift	276		167		44	
Intergenic_region	563,630		326,261		175,014	
Intron	916,272		576,268		191,182	
Missense	3520		5717		1667	
Non_coding_transcript_exon	5848		3846		2091	
Non_coding_transcript	18		10		1	
Protein_protein_contact	120		17		5	
Sequence_feature	7978		5930		1457	
Splice_acceptor	80		131		21	
Splice_donor	56		138		17	
Splice_region	1313		1174		437	
Start_lost	16		14		3	
Stop_gained	58		378		29	
Stop_lost	25		4		9	
Structural_interaction	1160		808		1	
Synonymous	2402		2727		81	
Upstream_gene	107,281		57,447		1301	

Common germline variants were excluded and variants were further filtered using SnpSift, with a total depth threshold at 30 (DP ≥ 30) and a minimum variance confidence of 200 (QUAL ≥ 200), and annotated by SnpEff. SNV, single nucleotide variant; UTR, untranslated region.

In particular, we noted that LNCaP had a larger number of stop_gained events, which are changes predicted to confer nonsense mutations and result in nonfunctional proteins or proteins with reduced function (Table 1). In LNCaP, SNVs and indel variants contributed 378 stop_gained events in 209 genes. We next identified biological processes overrepresented in this gene set (Table 2). This included a C→T transition at amino acid position 318 of menin (*MEN1*) (c.T954A in NCBI RefSeq NM_000244). Somatic inactivating mutations of menin are found in endocrine cancers (Falchetti *et al.* 2009), suggesting that *MEN1* is a tumor suppressor gene. However, it has recently been reported that *MEN1* is an oncogene in prostate cancer. Menin interacts with the androgen receptor and patients with overexpression of *MEN1* show poor OS (Malik *et al.* 2015). The *MEN1* SNV is present in an LNCaP sample interrogated by whole-exome sequencing (Taylor *et al.* 2010). Therefore, it is not likely to be a sequencing or data processing artifact. The functional regions of menin are currently not known, thus, the effect of the LNCaP premature stop codon event cannot be predicted. Interrogation of eight cBioPortal data sets suggests that inactivating mutations in the coding sequence of *MEN1* in prostate cancer is unique to LNCaP (data not shown); however, it is possible that distinct patient populations possess this variant (*e.g.*, see Lindquist *et al.* (2016)).

**Table 2:** Significantly overrepresented biological processes associated with sequence variants contributing stop_gained events in the PC3 and LNCaP prostate cancer cell lines

GO Term	Genes	Fisher’s Exact *P*
PC3 private sequence variants		
Detection of bacterium	*HLA-DRB1*, *HLA-A*, *HLA-DRB5*, *HLA-B*	5.1E−10
Antigen processing and presentation	*HLA-DRB1*, *HLA-A*, H*LA-DRB5*, *HLA-B*	2.4E−07
Interferon-γ-mediated signaling pathway	*HLA-DRB1*, *HLA-A*, *HLA-DRB5*, *HLA-B*	6.7E−07
Immune response	*HLA-DRB1*, *HLA-A*, *HLA-DRB5*, *HLA-B*, *IL1A*	4.7E−05
Antigen processing and presentation of endogenous peptide antigen via MHC class I via ER pathway, TAP-independent	*HLA-A*, *HLA-B*	2.9E−06
Regulation of interleukin-10 secretion	*HLA-DRB1*, *HLA-DRB5*	2.9E−06
Regulation of interleukin-4 production	*HLA-DRB1*, *HLA-DRB5*	5.8E−06
Protection from natural killer cell mediated cytotoxicity	*HLA-A*, *HLA-B*	9.6E−06
Immunoglobulin production involved in immunoglobulin mediated immune response	*HLA-DRB1*, *HLA-DRB5*	9.6E−06
Humoral immune response mediated by circulating immunoglobulin	*HLA-DRB1*, *HLA-DRB5*	2E−05
Antigen processing and presentation of exogenous peptide antigen via MHC class I, TAP-independent	*HLA-A*, *HLA-B*	3.5E−05
T-helper 1-type immune response	*HLA-DRB1*, *HLA-DRB5*	6.3E−05
Positive regulation of T cell mediated cytotoxicity	*HLA-A*, *HLA-B*	7.5E−05
Inflammatory response to antigenic stimulus	*HLA-DRB1*, *HLA-DRB5*	0.0001
Antigen processing and presentation of peptide or polysaccharide antigen via MHC class II	*HLA-DRB1*, *HLA-DRB5*	0.00013
Negative regulation of interferon-γ production	*HLA-DRB1*, *HLA-DRB5*	0.00036
Positive regulation of insulin secretion involved in cellular response to glucose stimulus	*HLA-DRB1*, *HLA-DRB5*	0.00039
Antigen processing and presentation of peptide antigen via MHC class I	*HLA-A*, *HLA-B*	0.00041
Negative regulation of T cell proliferation	*HLA-DRB1*, *HLA-DRB5*	0.00063
Protein tetramerization	*HLA-DRB1*, *HLA-DRB5*	0.00074
Antigen processing and presentation of exogenous peptide antigen via MHC class I, TAP-dependent	*HLA-A*, *HLA-B*	0.0018
Type I interferon signaling pathway	*HLA-A*, *HLA-B*	0.0019
T cell costimulation	*HLA-DRB1*, *HLA-DRB5*	0.0028
Antigen processing and presentation of exogenous peptide antigen via MHC class II	*HLA-DRB1*, *HLA-DRB5*	0.0038
Protein deubiquitination	*USP17L18*, *USP17L11*	0.0044
LNCaP private sequence variants		
Bundle of His cell to Purkinje myocyte communication	*GPR155*, *GNAS*, *CBR3*, *CHL1*	0.00012
Response to nitrosative stress	*PRKCQ*, *CD3E*, *NLRP3*	0.00068
Cognition	*FPGT-TNNI3K*, *TNNI3K*	0.0008
Interleukin-1 β production	*MEN1*, *NTRK3*, *PAX6*, *PRKDC*	0.0011
Positive regulation of interleukin-4 production	*LAMA2*, *PRKCQ*, *ROBO1*, *PAX6*, *SPTBN1*, *CHL1*	0.0012
Humoral immune response mediated by circulating immunoglobulin	*GCLC*, *DUSP6*	0.0023
Type B pancreatic cell differentiation	*NTRK3*, *AP1B1*, *FREM2*, *ROBO1*, *PRKDC*, *TBC1D32*	0.004
Negative regulation of protein phosphorylation	*LRP1*, *STAB1*, *VTN*, *SSC4D*, *AMN*, *DMBT1*	0.0042
Axon guidance	*NLRP3*, *CASP1*	0.0076
Heart development	*LAMA2*, *FREM2*, *ROBO1*, *STAB1*, *ITGB4*, *VTN*, *CERCAM*, *COL16A1*, *CHL1*, *AOC3*	0.014
Receptor-mediated endocytosis	*EXO1*, *HLA-DQB1*	0.016
Cell adhesion	*MEN1*, *PAX6*	0.027
Shared sequence variants (PC3 and LNCaP)		
Sensory perception of taste	*TAS2R43*, *TAS2R31*	0.00048
Detection of chemical stimulus involved in sensory perception of bitter taste	*TAS2R43*, *TAS2R31*	0.00092
*O*-glycan processing	*MUC3A*, *MUC6*	0.0021
Digestion	*PRSS2*, *PRSS1*	0.0023
Extracellular matrix disassembly	*PRSS2*, *PRSS1*	0.0033

Stop_gained events are denoted changes predicted to confer nonsense mutations and result in nonfunctional proteins or proteins with reduced function. Gene enrichment analysis was performed using DAVID (Database for Annotation, Visualization and Integrated Discovery). MHC, major histocompatibility complex; ER, endoplasmic reticulum; TAP, transporter associated with antigen processing.

**Figure 2:**
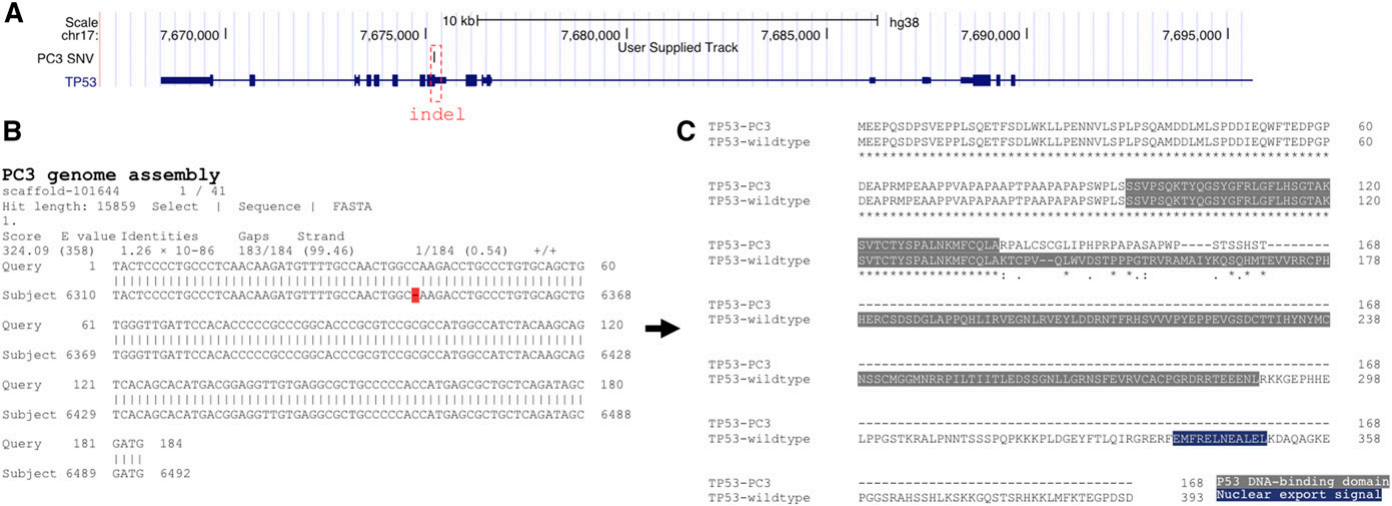
Overview of a p53 (*TP53*) sequence variant in the PC3 prostate cancer cell line. (A) Genome browser display showing an indel event in the PC3 p53 gene (*TP53*). (B) Sequence alignment of *TP53* in the PC3 genome and the reference genome assembly (Ensembl GRCh38 build 82). An indel is indicated in red. (C) Sequence alignment of *TP53* protein products encoded by PC3 and the reference transcript NM_000546. An indel results in a frameshift (p.A138fs) and a truncated protein in PC3. chr, chromosome; SNV, single nucleotide variation.

In PC3, 58 stop_lost events (Table 1) in 20 genes, (*AHNAK2*, *DNAH6*, *FAT3*, *GOLGA6L3*, *GOLGA6L9*, *HLA-A*, *HLA-B*, *HLA-DRB1*, *HLA-DRB5*, *HOXA9*, *IL1A*, *ITPR2*, *MEGF6*, *MUC19*, *OR8K3*, *PRPF3*, *PRSS1*, *PTPRD*, *USP17L11*, and *USP17L18*) were observed. There was a significant enrichment for HLA class II antigen presenting genes associated with the immune response (Table 3; Fisher’s exact *P* ∼ 0.05). It has recently been shown that the PC3, LNCaP, and DuPro (but not the DU145) prostate cancer cell lines and prostate cancer tissues express HLA class II molecules (Younger *et al.* 2008; Doonan and Haque 2015). However, we could not identify any prostate cancer patients with stop_lost events in these genes using the cBioPortal tool (Cerami *et al.* 2012; Gao *et al.* 2013) (data not shown). While evasion of the antitumour immune response is an emerging research area (Drake 2010; Corrales *et al.* 2016), caution should be exercised when considering the use of PC3 cells in these studies. Sequence variant analysis and interrogation of the PC3 *de novo* genome assembly by BLAST and GMAP confirmed that the tumor suppressor p53 (*TP53*) is inactivated by a single frameshift event (p.A138fs; indel; c.*4955A in NCBI RefSeq NM_000546) (Carroll *et al.* 1993) (Figure 2A).

**Table 3:** Putative deleted genes and their expression in the LNCaP and PC3 prostate cancer cell lines

Symbol	Description	CNV Region	Gene Start	Gene End	NP UPC	LNCaP UPC	PC3 UPC
LNCaP							
* PWRN1*	Prader-Willi region nonprotein coding RNA 1	15:24430001–24520000	24493137	24652130	0.0	0.0	0.0
PC3							
* ATP6V0A1*	ATPase H+ transporting V0 subunit a1	17:42110001–42520000	42458844	42522611	1	1	0
* CDH18*	Cadherin 18	5:19850001–19960000	19472951	20575873	0	0	0
* CIC*	Capicua transcriptional repressor	19:42280001–42320000	42268537	42295797	1	1	0
* CTNNA1*	Catenin α 1	5:138770001–138980000	138610967	138935034	1	1	0
* DDX3Y*	DEAD-box helicase 3, Y-linked	Y:11530001–16450000	12904108	12920478	1	1	0
* DHX58*	DExH-box helicase 58	17:42110001–42520000	42101404	42112733	1	0	0
* DYDC1*	DPY30 domain containing 1	10:80250001–80560000	80336105	80356755	0	0	0
* DYDC2*	DPY30 domain containing 2	10:80250001–80560000	80344745	80368073	0	0	0
* FAM213A*	Family with sequence similarity 213 member A	10:80250001–80560000	80407829	80437115	1	0	0
* GHDC*	GH3 domain containing	17:42110001–42520000	42188799	42194532	1	1	0
* HCRT*	Hypocretin neuropeptide precursor	17:42110001–42520000	42184060	42185452	0	0	0
* HSPB9*	Heat shock protein family B (small) member 9	17:42110001–42520000	42121431	42123352	0	0	0
* KAT2A*	Lysine acetyltransferase 2A	17:42110001–42520000	42113108	42121358	1	1	0
* KCNH4*	Potassium voltage-gated channel Subfamily H member 4	17:42110001–42520000	42156891	42181278	0	0	0
* LIPJ*	Lipase family member J	10:87910001–88750000	88586753	88606976	0	0	0
* LRRTM2*	Leucine rich repeat transmembrane neuronal 2	5:138770001–138980000	138868923	138875368	0	0	0
* MAT1A*	Methionine adenosyltransferase 1A	10:80250001–80560000	80271820	80289684	0	0	0
* MIR548AT*	MicroRNA 548at	17:42110001–42520000	42494773	42494830	0	0	0
* NLGN4Y*	Neuroligin 4, Y-linked	Y:11530001–16450000	14522638	14845650	1	0.2	0
* PAFAH1B3*	Platelet activating factor acetylhydrolase 1b catalytic subunit 3	19:42280001–42320000	42297033	42303546	1	0.7	0
* PRR19*	Proline rich 19	19:42280001–42320000	42302098	42310821	0	0	0
* PTEN*	Phosphatase and tensin homolog	10:87910001–88750000	87863113	87971930	1	1	0
* PTRF*	Polymerase I and transcript release factor	17:42110001–42520000	42402452	42423517	1	0	0
* RAB5C*	RAB5C, member RAS oncogene family	17:42110001–42520000	42124976	42155044	1	1	0
* RNLS*	Renalase, FAD dependent amine oxidase	10:87910001–88750000	88273864	88584530	0.6	0	0
* SH2D4B*	SH2 domain containing 4B	10:80250001–80560000	80537902	80646560	0	0	0
* SIL1*	SIL1 nucleotide exchange factor	5:138770001–138980000	138946720	139293557	1	1	0
* SIRPB1*	Signal regulatory protein β 1	20:1580001–1620000	1563521	1620061	0	0	0
* STAT3*	Signal transducer and activator of transcription 3	17:42110001–42520000	42313324	42388568	1	1	0
* STAT5A*	Signal transducer and activator of transcription 5A	17:42110001–42520000	42287547	42311943	1	0	0
* STAT5B*	Signal transducer and activator of transcription 5B	17:42110001–42520000	42199168	42276707	1	1	0
* MEM145*	Transmembrane protein 145	19:42280001–42320000	42313325	42325062	0	0	0
* TMSB4Y*	Thymosin β 4, Y-linked	Y:11530001–16450000	13703567	13706024	0	0	0
* TSPAN14*	Tetraspanin 14	10:80250001–80560000	80454166	80533123	1	1	0
* TSPY1*	Testis-specific protein, Y-linked 1	Y:9450001–10200000	9466955	9490081	0	0	0
* TTTY13*	Testis-specific transcript, Y-linked 13 (nonprotein coding)	Y:21420001–21630000	21583600	21594666	0	0	0
* TTTY15*	Testis-specific transcript, Y-linked 15 (nonprotein coding)	Y:11530001–16450000	12662334	12692233	1	1	0
* USP9Y*	Ubiquitin specific peptidase 9, Y-linked	Y:11530001–16450000	12701231	12860839	1	1	0
* UTY*	Ubiquitously transcribed tetratricopeptide repeat containing, Y-linked	Y:11530001–16450000	13248379	13480673	1	1	0

CNV regions are listed for putative homozygous deletion events (CNV = 0). UPC refers to Universal exPression Code score, where UPC value > 0.5 indicates that a gene is actively transcribed. CNV, copy number variation; NP, normal prostate tissue; RAB5C, Ras-related protein Rab-5C; FAD, flavin adenine dinucleotide.

PC3 shared 0.26 M sequence variants (166,912 SNVs and 89,919 indels) with LNCaP, and 21 of these constituted stop_lost events (Table 1). Overrepresented biological processes in PC3 and LNCaP included “*O*-glycan processing” (the mucins *MUC3A* and *MUC6*) and “extracellular matrix disassembly” (the trypsinogens *PRSS1* and *PRSS2*) (Table 2). Interestingly, while we have identified *MUC3A* stop_gained events in PC3 and LNCaP, cell lines generated from Caucasian patients, a recent study suggests that MUC3A protein-changing variants are rare in Caucasians and predominant in African Americans, the subpopulation with the highest prevalence of prostate cancer, where MUC3A changes are observed in 88% of patients (Lindquist *et al.* 2016).

Taken together, these data indicate that protein-coding genes in LNCaP are perturbed extensively by point and indel mutations. Even after filtering steps, our LNCaP data (at passage four from the ATCC stock) reveal a clear difference in the number of particular variant events compared to PC3. However, previous exome sequencing work suggests that the genome of the parental LNCaP strain sequenced here (clone FGC) and its derived strains are inherently unstable (Spans *et al.* 2012, 2014), and this could give rise to the apparently high mutation rate in protein-coding sequences. As with studies of the HeLa genome (Adey *et al.* 2013; Landry *et al.* 2013), further genome sequencing efforts are warranted to investigate whether the variants reported here are somatic mutations found in particular LNCaP strains, or if they represent preexisting subpopulations within the parental LNCaP strain. In the future, single-cell WGS is likely to resolve this issue. Nevertheless, LNCaP and PC3 appear to have distinct SNV and indel profiles.

### Putative gene loss

Most human cancers have CNVs, which impact upon gene dosage through loss or gain of whole chromosomes or chromosome segments (Hanahan and Weinberg 2011). Previous studies have described CNVs in PC3 and LNCaP using targeted techniques, such as exome sequencing. However, WGS, together with continuously updated gene annotations, offers improved detection of copy number changes (Meynert *et al.* 2014; Belkadi *et al.* 2015; Warr *et al.* 2015).

CNVs were identified using the R package cn.mops (Klambauer *et al.* 2012). In particular, we wished to identify genes that are lost in PC3 and LNCaP. The absence of this information can misinform even the most well-designed *in vitro* or cell line xenograft experiment (*e.g.*, where a gene in an important pathway is lost). In the context of CNV analysis, we were interested in identifying putative homozygous deletions (CNV = 0; CNV0 events), *i.e.*, genes that are inactivated by partial or complete gene deletion. To inform this analysis, we also considered the transcriptional potential of each gene by analyzing publicly available transcriptome (RNA-seq) data from normal prostate, LNCaP, and PC3. Genes with a UPC value of ∼0.5 were considered inactive (Piccolo *et al.* 2013).

Although a large number of SNVs and indel variations were observed in LNCaP, only a single homozygous deletion event (CNV0) was observed in this cell line. In the complex Prader-Willi gene locus there was a putative loss of *PWRN1*, a gene associated with epigenetic reprogramming during spermatogenesis (Wawrzik *et al.* 2009) (Table 3).

In contrast to LNCaP, 39 CNV0 events were found in PC3 (Table 3). CNV of the Y chromosome was evident from the PC3 sequence coverage (Figure 1A). In agreement with previous studies employing cDNA microarrays (Clark *et al.* 2003) and multicolor fluorescence *in situ* hybridization (Aurich-Costa *et al.* 2001), our CNV analysis revealed that large regions of the Y chromosome (including eight genes) were deleted in PC3 (Table 3). Several genes on chromosome 5 (*CDH18*, *CTNNA1*, *LRRTM2*, and *SIL1*), chromosome 10 (*DYDC1*, *DYDC2*, *FAM213A*, *LIPJ*, *MAT1A*, *PTEN*, *RNLS*, *SH2D4B*, and *TSPAN14*), and chromosome 17 (*ATP6V0A1*, *DHX58*, *GHDC*, *HCRT*, *HSPB9*, *KAT2A*, *KCNH4*, *MIR548AT*, *PTRF*, *RAB5C*, *STAT3*, *STAT5A*, and *STAT5B*) have also previously been reported to be deleted in PC3 (Liu *et al.* 2008; Krohn *et al.* 2012; The Cancer Genome Atlas Research Network 2015; Ibeawuchi *et al.* 2015).

Clinical observations and experimental studies indicate that the growth hormone receptor (GHR) mediates the development and progression of cancer (Brooks and Waters 2010), and *GHR* expression is elevated in prostate cancer cell lines and tissues (Chopin *et al.* 2002; Weiss-Messer *et al.* 2004). Interestingly, we noted that the genes encoding the classical growth hormone receptor signaling molecules STAT3 (*STAT3*) and STAT5 (*STAT5A* and *STAT5B*) were lost in PC3 cells. Thus, autocrine GHR actions are likely to be associated with alternative signaling pathways (Barclay *et al.* 2010) in PC3. Loss of *STAT3* in PC3 has been firmly established experimentally (Yuan *et al.* 2005; Pencik *et al.* 2015), and there is evidence to suggest that STAT3 suppresses prostate cancer metastasis and confers a good prognosis (Pencik *et al.* 2015).

**Figure 3:**
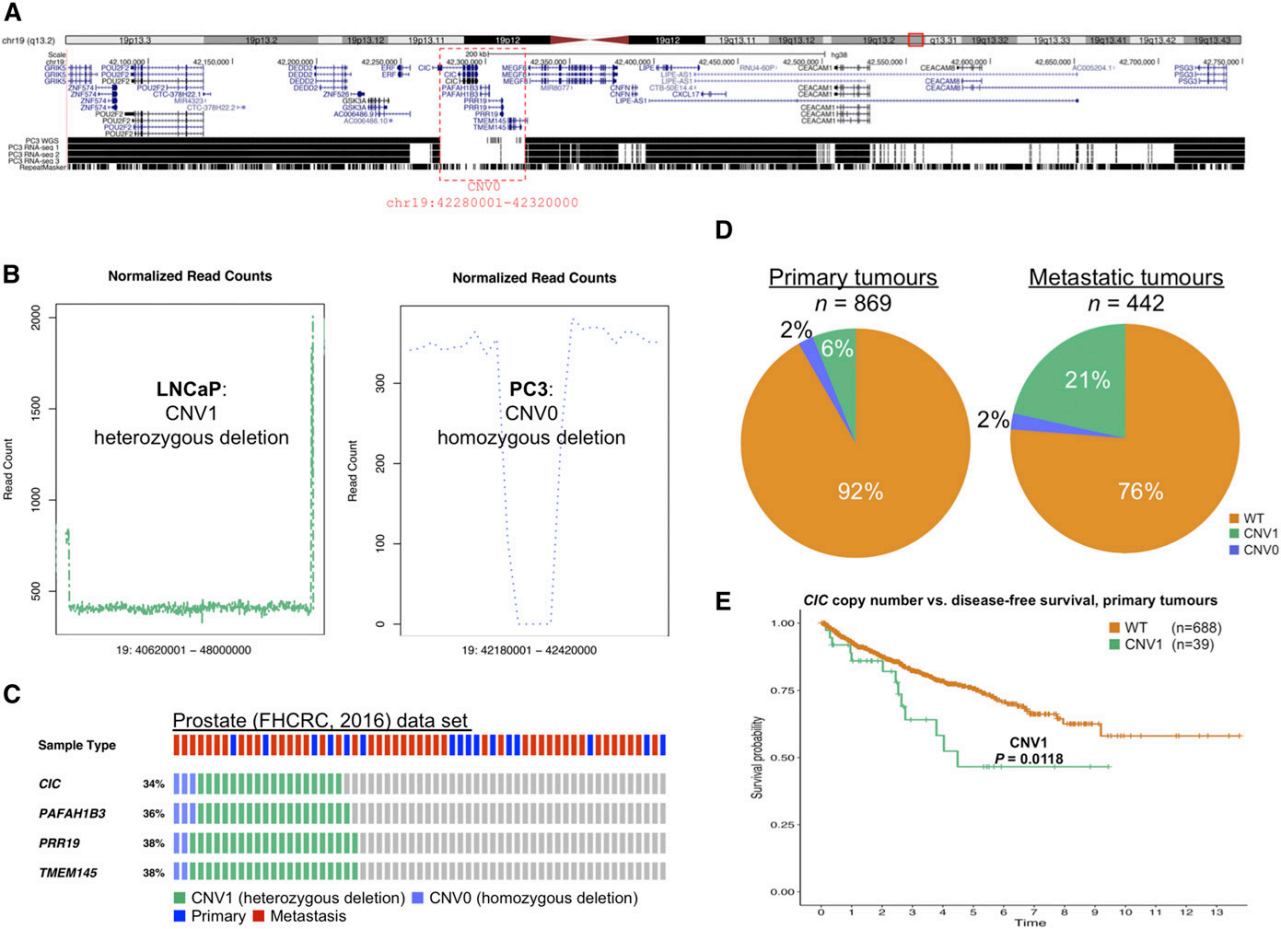
Deletion of the tumor suppressor Capicua (*CIC*) in prostate cancer (A) Genome browser display showing a CNV0 event (red dotted line) on chromosome 19 that spans *CIC*, *PAFAH1B3*, *PRR19*, and *TMEM145* in the PC3 prostate cancer cell line. (B) Plot of putative chromosomal loss spanning the four-gene region in LNCaP (left panel) and PC3 (right panel). The *x*-axis represents the genomic position and the *y*-axis the number of normalized counts. (C) Loss of the chromosome 19 region encompassing *CIC*, *PAFAH1B3*, *PRR19*, and *TMEM145* in the ‘Prostate FHCRC 2016’ cBioPortal data set. Individual tumor samples are shown in columns and genes in rows. (D) *CIC* copy number alterations in primary and metastatic prostate cancer samples from eight clinical data sets interrogated using cBioPortal. (E) Disease-free survival in primary prostate cancer patients with loss of a single *CIC* gene copy (*n* = 13 relapse events) is significantly decreased compared to those without any *CIC* deletion events. ‘The Prostate (MSKCC 2010)’, ‘Prostate (MSKCC 2014)’, and ‘Prostate (TCGA)’ cBioPortal data sets were interrogated. *P*-values were calculated by Kaplan–Meier analysis (log-rank test). Time denotes years. CNV0, homozygous deletion; CNV1, heterozygous deletion; WT, wild-type.

We identified a homozygous deletion event spanning four genes (*CIC*, *PAFAH1B3*, *PRR19*, and *TMEM145*) on chromosome 19 in PC3 (Figure 3A). In LNCaP, a genome coverage plot of reads flanking this region revealed a putative heterozygous event (CNV1; loss of a single copy of the same genes) (Figure 3B). Of these four genes, the mammalian homolog of *Drosophila* *CIC* (Jiménez *et al.* 2012) is particularly interesting. Capicua is a transcriptional repressor of cancer metastasis in a number of cancers (Choi *et al.* 2015; Okimoto *et al.* 2017). Recent WGS data also suggests that *CIC* is lost in PC3 cells (Iorio *et al.* 2016). Homozygous deletions of *CIC* have been reported in neuroblastoma (Nagaishi *et al.* 2014; Fransson *et al.* 2016), and a homozygous deletion of *CIC* in a subpopulation of H1975 human nonsmall cell lung cancer cell line xenografts rendered them highly metastatic (Okimoto *et al.* 2017). We interrogated 75 cBioPortal data sets from diverse tumors, confirming that one or two copies of *CIC* are lost in many cancer types (see Supplemental Material, Figure S1).

*CIC* is abundantly expressed in normal prostate tissue, whereas its expression is reduced in primary tumors and ablated in metastatic prostate cancer (Choi *et al.* 2015). To characterize the potential clinical significance of *CIC* deletions in prostate cancer, we further examined 1311 tumors from eight data sets using the cBioPortal tool. While homozygous deletion events of the four genes deleted in PC3 cells were rare, a substantial fraction of prostate tumors harbored heterozygous deletions of these genes (Figure 3C). Approximately 6% of primary prostate tumors had heterozygous deletions and 2% had homozygous deletions of *CIC*, whereas 21% of metastatic tumors had homozygous *CIC* deletions and 2% heterozygous deletions (Figure 3D).

Prostate cancer relapse or recurrence frequently results in incurable metastasis, ultimately causing patient death (Wu *et al.* 2014; Weiner *et al.* 2016). As *CIC* deletions were more frequent in metastatic tumors, we reasoned that deletion of one or both copies of *CIC* is a means by which primary tumors in patients that eventually develop metastatic lesions achieve increased fitness and survival. The association between *CIC* homozygous deletion events and DFS in primary tumors could not be reliably assessed due to the low number (*n* = 2) of patients with recorded relapses; however, patients with primary prostate tumors with one lost copy of *CIC* (heterozygous deletion events) had a significantly worse outcome (*P* = 0.018, log-rank test) (Figure 3E). Similarly, OS is significantly worse in advanced-stage gastric cancer patients with low *CIC* expression (Okimoto *et al.* 2017).

A recent study comparing PC3 and LNCaP reported that the long form of the CIC protein (CIC-L) was not expressed and that the short form (CIC-S) was expressed at extremely low levels in PC3 cells (Choi *et al.* 2015). Our CNV analysis, employing WGS reads, interrogation of the *de novo* PC3 assembly using BLAST and GMAP, and analysis of RNA-seq reads mapped to the reference genome, failed to detect an intact *CIC* gene in PC3. We sequenced low-passage PC3 cells sourced directly from ATCC and speculate that the previous study (Choi *et al.* 2015) detected low-level gene expression by PC3 subpopulations with intact *CIC* resulting from genetic drift during prolonged subculture (passaging; see Festuccia *et al.* (1999); Li *et al.* (2008)).

Taken together, these data suggest that although a rare event in prostate tumors, homozygous deletion of *CIC* is not an idiosyncrasy of the PC3 cell line. Moreover, loss of a single gene copy of *CIC* is relatively common in prostate cancer. We speculate that disruption of one or both copies of *CIC* renders prostate cancer patients susceptible to an adverse disease outcome. A previous study employing forced overexpression of *CIC* in PC3 and LNCaP demonstrated that *CIC* is repressed by a trio of microRNAs (Choi *et al.* 2015). Altered MAPK signaling through the ERK pathway also suppresses endogenous *CIC* in lung cancer (Okimoto *et al.* 2017). Collectively, our data raise the possibility that the combination of microRNA repression, altered ERK signaling, and somatic events in the *CIC* locus promote tumorigenesis and confer a poor disease outcome.

### Relevance of findings

In summary, we provide genome sequence data for PC3 and LNCaP, prostate cancer cell lines commonly employed in cancer research.

These data contribute to a catalog of cancer genomes, adding to recent whole-transcriptome sequencing, pharmacological profiling, and whole-exome sequencing efforts (Barretina *et al.* 2012; Klijn *et al.* 2015; Iorio *et al.* 2016) aimed at enhancing our understanding of human disease. For example, the phenomenon of androgen independence in prostate cancer has intrigued scientists for decades. Of the two cell lines interrogated in our study, PC3 is androgen-independent, whereas the LNCaP strain sequenced (LNCaP-FGC) is androgen-dependent. Recent work, including an investigation of 150 patients with metastatic CRPC (Robinson *et al.* 2015), suggests that anomalies (mutations, amplifications, and deletions) in a number of genes in the androgen receptor pathway play a role in the transition to androgen independence. We speculate that future work—employing WGS, RNA-sequencing, epigenetic profiling, and similar high-throughput methods—on a large number of cell lines and clinical samples is likely to identify genes critical for androgen independence. For instance, an androgen-independent strain of LNCaP (LNCaP-LNO) has been developed from cultures of an early passage of the LNCaP cells sequenced in our study (LNCaP-FGC) (van Steenbrugge *et al.* 1991). LNCaP-LNO and LNCaP-FGC were compared at the gene expression level (Oosterhoff *et al.* 2005); hinting that specific gene mutations or copy number events render LNCaP-LNO cells androgen-insensitive.

Raw reads (see *Data availability* in *Materials and Methods*) and sequence (SNV and indel) and CNV data are made available. We have generated *de novo* genome assemblies of both cell lines, allowing genes of interest to be investigated further, enabling, for example, the validation of gene loci associated with novel transcripts obtained from Trinity *de novo* transcriptome analysis (Grabherr *et al.* 2011; Haas *et al.* 2013). In addition, the genomes can be interrogated using a BLAST server, available at http://ghrelinlab.org. We acknowledge the limitations of short-insert (350 bp) genome sequencing, particularly when resolving complex repetitive or heterozygous regions (Rhoads and Au 2015; Merker *et al.* 2016). However, we anticipate that as sequencing becomes increasingly affordable, our sequencing efforts will complement future long-read genome assembly work and prove useful when correcting for errors (sequence polishing).

Finally, we reveal that one or both copies of *CIC*, a tumor metastasis suppressor gene, are frequently lost in prostate cancer and could drive metastatic CRPC. We anticipate that further biological insights into the role of Capicua in prostate cancer will shortly be gained by the research community, in line with the ethos of *G3: Genes*, *Genomes*, *Genetics* Genome Reports.

## Supplementary Material

Supplemental material is available online at www.g3journal.org/lookup/suppl/doi:10.1534/g3.117.039909/-/DC1.
