# Supplementary material for: Whole-Genome Sequence of the Metastatic PC3 and LNCaP Human Prostate Cancer Cell Lines
Source: G3 (Bethesda). 2017 Apr 14;7(6):1731–41. doi: 10.1534/g3.117.039909 (PMC5473753; doi:10.1534/g3.117.039909)
Supplement: Supplementary file 1 [file 1731File002.pdf]

Supplemental Material

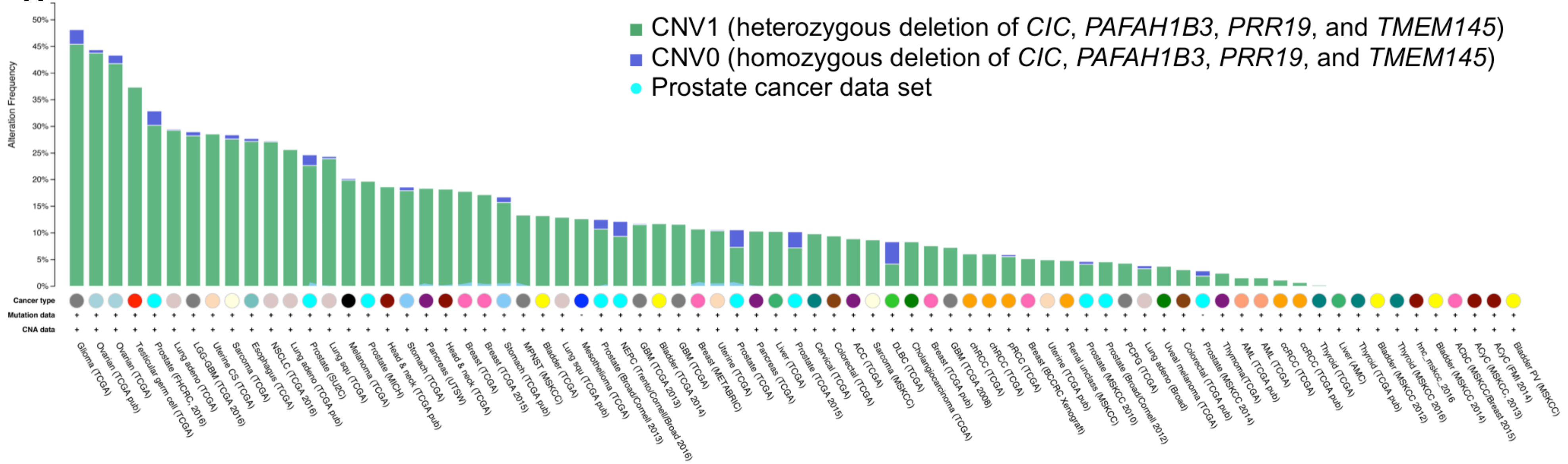

Figure S1. Histogram of homozygous (CNV0) and heterozygous (CNV1) gene deletion events, associated with four neighbouring genes (*CIC*, *PAFAH1B3*, *PRR19*, and *TMEM145*), in diverse cancers. The y-axis show the percentage of tumours that have an alteration in the four genes (CNV0 events shown in blue; CNV1 events in green), and the x-axis denote a cBioPortal data set (prostate cancer data sets are indicated in turquoise).
